# The Complete Chloroplast Genome of *Ficus gasparriniana* var. *laceratifolia* Reveals Discordance Between Morphology-Based Classification and Plastid Phylogeny

**DOI:** 10.3390/genes17080871

**Published:** 2026-07-26

**Authors:** Yong Shi, Jiejun Liu, Lei Ren, Chen Feng, Yuan Liu

**Affiliations:** 1 Jiangxi Provincial Key Laboratory of Ex Situ Plant Conservation and Utilization, Lushan Botanical Garden, Chinese Academy of Sciences, Jiujiang 332900, China; 2 School of Life Sciences, Nanchang University, Nanchang 330031, China

**Keywords:** chloroplast genome, codon usage, *Ficus*, hypervariable regions, inverted repeat boundary, Moraceae, plastome phylogeny, RSCU

## Abstract

**Background/Objectives:** *Ficus gasparriniana* var. *laceratifolia* (H. Lév. & Vaniot) Corner is treated as a variety of *F. gasparriniana* and placed in *Ficus* subg. *Ficus* on morphological grounds, but complete plastome evidence for its plastid phylogenetic placement has been lacking. We assembled and analyzed its chloroplast genome to evaluate this morphology-based placement using plastid genomic evidence and to expand genomic resources for the genus. **Methods:** The plastome was assembled from paired-end reads using GetOrganelle and subsequently annotated. We characterized its genome architecture, simple sequence repeats (SSRs), codon-usage bias, inverted repeat (IR) junctions, and nucleotide diversity after standardizing sequence start positions and small single-copy (SSC) region orientation. Plastid phylogenetic relationships were inferred from four single-IR datasets: whole-plastome, coding, non-coding, and partitioned. **Results:** The 160,476-bp plastome exhibited the typical quadripartite structure and contained 110 unique genes. Its repeat composition and preference for A/U-ending codons were consistent with an AT-rich plastome, and 61 SSRs and five candidate variable regions represented potential marker resources for future *Ficus* studies. In all four phylogenetic datasets, *F. gasparriniana* var. *laceratifolia* was consistently grouped with *F. pumila* with maximum ultrafast bootstrap support (UFBoot = 100), conflicting with its morphology-based classification. Approximately unbiased (AU) tests rejected the sampled morphology-based constraint in every dataset, indicating that this morphology–plastid discordance was robust to dataset choice. **Conclusions:** This plastome provides a valuable genomic resource and reveals robust discordance between morphology-based classification and plastid phylogenetic placement. These findings provide a foundation for future nuclear-genomic and population-level tests of the alternative evolutionary scenarios underlying this discordance.

## 1. Introduction

Morphology-based classifications do not always align with the phylogenetic signal recovered from plastid or nuclear genomes in *Ficus* L., the largest genus in Moraceae [1,2]. This tension is consequential because infrageneric classification in *Ficus* has long rested on morphological characters—habit, branching architecture, leaf morphology, syconium position and size, and reproductive traits [1,3,4]. Yet molecular phylogenetic studies have repeatedly documented cases in which these morphology-based hypotheses conflict with plastid or nuclear relationships [2,5,6,7], indicating that genomic data can provide an independent line of evidence for re-examining traditional taxonomic assignments.

Chloroplast genomes are a particularly informative source of such evidence for plant systematics, comparative genomics, and molecular-marker development. Plastomes vary among taxa in gene content, repeat composition, codon usage, inverted-repeat (IR) boundary configuration, and nucleotide diversity, and this variation can resolve relationships among closely related lineages [8]. Because plastomes are typically inherited as single, largely non-recombining cytoplasmic units in angiosperms, plastome phylogenies trace a plastid lineage rather than the complete organismal history of a species—making them well suited to testing whether the plastid placement of a taxon agrees with its morphology-based assignment. In *Ficus*, plastome-scale studies have improved phylogenetic resolution and revealed instances in which plastid relationships diverge from morphology-based classifications or nuclear evidence [6,7,9], underscoring the value of complete plastomes for such tests.

**Figure 1:**
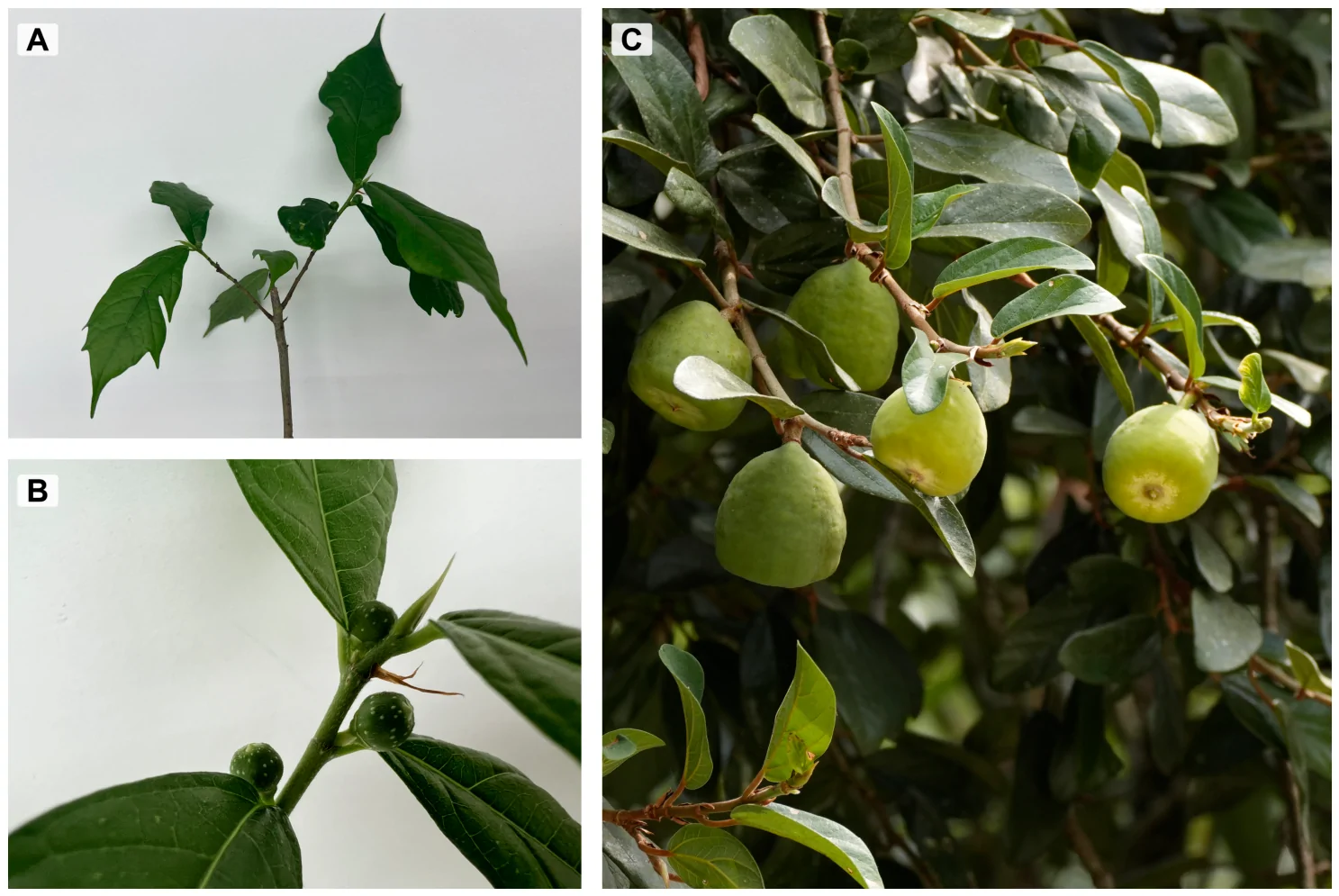
Morphological comparison of *Ficus gasparriniana* var. *laceratifolia* and *Ficus pumila*. (**A**) Vegetative branch and foliage of the focal plant. (**B**) Close-up showing small axillary syconia borne on normal branchlets. (**C**) *F. pumila*, showing foliage and larger syconia characteristic of its climbing/scandent habit. Photographs by Yuan Liu and Lei Ren.

*Ficus gasparriniana* var. *laceratifolia* offers a clear case for testing whether morphology-based classification agrees with plastid phylogenetic placement. Under the Flora of China treatment [10], it is treated as a variety of *F. gasparriniana* and placed in *Ficus* subg. *Ficus*. Morphologically, it has obovate leaves with a greenish-white, glabrous or sparsely pubescent abaxial surface, 1–4 irregular apical teeth, and 5–7 secondary veins on each side, distinguishing it from var. *gasparriniana* (densely hairy, pubescent leaf undersides) and var. *esquirolii* (lanceolate, entire leaves with 8–18 secondary veins on each side) [10]. Its shrub habit and small axillary syconia on normal leafy shoots also contrast sharply with those of *F. pumila* L. in subg. *Synoecia* (Figure 1), a climber or scandent plant with rooting sterile branchlets, dimorphic leaves, and substantially larger syconia [10]. Beyond this taxonomic interest, the variety is used in traditional medicine—like many other *Ficus* species used to treat inflammatory and gastrointestinal diseases [11]—and has been documented as an important wild resource in traditional medicinal diets and indigenous healthcare [12]. Because the genus also exhibits pronounced morphological convergence and reticulate evolutionary histories that complicate species delimitation [13], a complete plastome sequence is valuable both for molecular authentication of such medicinal material and for resolving its plastid relationships against this morphology-based placement. The new plastome further provides a genomic resource for taxonomic comparison, marker discovery, and conservation-genetic research.

Here, we asked whether the complete plastome of *F. gasparriniana* var. *laceratifolia* supports its morphology-based placement in subg. *Ficus* or instead reveals a different plastid relationship. To address this question, we assembled and annotated its complete chloroplast genome from high-throughput sequencing data. We characterized its genome structure, gene content, repeat composition, relative synonymous codon usage, IR boundary features, and nucleotide-diversity patterns relative to representative *Ficus* plastomes. We then reconstructed single-IR phylogenies using whole-plastome, coding, non-coding, and partitioned datasets. This study provides a complete plastome resource, identifies candidate variable regions for marker development, and enables a direct test of congruence between morphology-based classification and plastid phylogeny in *Ficus*.

## 2. Materials and Methods

### 2.1. Plant Material, DNA Extraction, and Sequencing

Fresh leaves were collected in Emeishan City, Sichuan Province, China (29.570512° N, 103.438255° E) and desiccated in silica gel. The material was identified morphologically as *F. gasparriniana* var. *laceratifolia* following the Flora of China treatment [10], and the determination was reviewed by both a plant taxonomist and a specialist in *Ficus*. A voucher specimen (LBG20261034) was deposited in the Herbarium of Lushan Botanical Garden, Chinese Academy of Sciences. Total genomic DNA was extracted from silica-dried leaf tissue using a modified CTAB protocol [14]. Paired-end sequencing (2 × 150 bp) was performed by BGI Genomics (Shenzhen, China) on a DNBSEQ-T7 sequencer. Raw reads were deposited under BioProject PRJNA1472929, BioSample SAMN60525120, and SRA accession SRR38916853.

### 2.2. Chloroplast Genome Assembly, Annotation, and Visualization

The raw library contained 19,209,689 paired-end read pairs, with Q20 and Q30 rates (percentages of bases with Phred quality scores ≥20 and ≥30) of 99.32% and 97.34%, respectively. To remove residual sequencing adapters and low-quality bases before assembly, the raw SRR38916853 reads were trimmed with Trimmomatic v0.39 [15]. The cleaned reads were then assembled de novo into a complete reference plastome for downstream comparative analyses using GetOrganelle v1.7.7.1 with the embplant_pt database [16], a maximum of 15 extension rounds (-R 15), and the default k-mer gradient (21, 55, 85, 115). Read support and plastome coverage were then assessed by mapping reads back to the assembly with BWA-MEM v0.7.19 [17] using a fixed batch size (-K 100000000); this mapping yielded 2,230,366 primary mapped reads (mapping rate 5.81%), and only primary alignments were used for coverage summaries. To confirm sequence closure and read support across the major structural junctions, circularity was evaluated from the GetOrganelle assembly graph and four synthetic junction references, each comprising 1-kb flanks on either side of the junction (2 kb total). A spanning read was defined as a primary alignment with a mapping quality (MAPQ) score ≥ 20 and at least 20 aligned bases on each side of the synthetic junction; unique spanning templates and proper-pair fragment bridges were counted separately (Table S8). Finally, coverage variation along the plastome was characterized using per-base coverage and mean coverage within 200-bp windows calculated with a custom script in R v4.4.2 [18] (Figure S1).

To obtain a reliable plastome annotation for structural and comparative analyses, the plastome was annotated with GeSeq [19], and all predicted transfer RNA (tRNA) genes were independently validated using tRNAscan-SE v2.0.12 [20]. Gene boundaries were manually curated against published *Ficus* annotations to check feature limits and annotation consistency. The circular plastome map and corresponding splicing diagrams were then generated with the CPGView web server [21] to summarize genome organization and display *cis*-splicing and *trans*-splicing gene structures.

To assess whether the morphology-based identification of *F. gasparriniana* var. *laceratifolia* was molecularly consistent with an independently sampled *F. gasparriniana* dataset, we retrieved publicly available whole-genome resequencing reads from SRR13279556 (BioProject PRJNA684963; BioSample SAMN17081732). The reads were assembled de novo using GetOrganelle v1.7.7.1 and SPAdes v4.2.0. Assembly completeness was evaluated using read support across the unresolved AT-rich region and genome-wide sequencing depth. The validated comparison assembly was normalized to the same reference start position and aligned with the focal plastome using MAFFT v7.525 [22]. Pairwise sequence identity and alignment differences were then quantified to evaluate plastome concordance between the two independently sampled materials.

### 2.3. SSR, Long-Repeat, and Codon Usage Analyses

To characterize repetitive elements as resources for future marker development, simple sequence repeats (SSRs) were identified in the *F. gasparriniana* var. *laceratifolia* plastome using MISA-web [23]. Minimum repeat thresholds were set to 10, 6, 5, 5, 5, and 5 repeat units for mono-, di-, tri-, tetra-, penta-, and hexanucleotide repeats, respectively. The maximum distance between two SSRs constituting a compound microsatellite was set to 100 bp.

For a comparison of long-repeat abundance across *Ficus*, forward and palindromic repeats were detected in a representative subset of six taxa: *F. gasparriniana* var. *laceratifolia*, *F. pumila*, *Ficus carica*, *Ficus racemosa*, *Ficus microcarpa*, and *Ficus lyrata*. These taxa were selected to include the focal variety, the key phylogenetic comparator *F. pumila*, sampled subg. *Ficus* representatives, and additional available *Ficus* plastomes. Repeats were identified using Vmatch v2.3.1 with a minimum repeat length of 30 bp and a Hamming distance of 3 [24].

To characterize synonymous codon-usage patterns in the newly assembled plastome, protein-coding CDS were extracted from the annotated GenBank record. To avoid overweighting genes duplicated in the inverted repeats, the primary analysis retained a single copy of each CDS and excluded pseudogenes, terminal stop codons, and incomplete terminal residues. Relative synonymous codon usage (RSCU), calculated as the ratio of the observed codon count to the count expected under equal use of synonymous codons for each amino acid [25], was then used to identify preferred codons and assess nucleotide-ending bias. As a sensitivity check on IR-copy treatment, the analysis was repeated using all annotated CDS, including duplicated IR copies (Table S3b).

### 2.4. Inverted Repeat Boundary Comparison

To compare inverted-repeat (IR) boundary structure among representative *Ficus* plastomes, four junctions involving the large single-copy (LSC), small single-copy (SSC), and IR regions (LSC/IRb, IRb/SSC, SSC/IRa, and IRa/LSC) were examined in six taxa using IRscope [26], with *F. gasparriniana* var. *laceratifolia* as the reference. Because inconsistent public annotations can be mistaken for genuine IR expansion or contraction, the GenBank records were first checked for start-position consistency, IR annotation completeness, and SSC orientation. Where annotations were incomplete, both IR copies were verified and their coordinates defined by self-BLAST using BLAST+ v2.17.0 [27] with the parameters -dust no -soft_masking false -perc_identity 80 -max_hsps 5000. To standardize inputs for direct IR-boundary comparison, repeat_region features were then added at the BLAST-supported coordinates, and SSC orientation was harmonized where necessary.

### 2.5. Nucleotide Diversity and Hypervariable-Region Detection

To identify candidate hypervariable regions for future species-level studies, a set of 13 *Ficus* plastomes was compared. To ensure homologous alignment of circular genomes, all sequences were normalized to the PZ482414.1 start position and SSC orientation, then aligned using MAFFT v7.525 with the parameters --auto --thread -1. The dataset comprised *F. gasparriniana* var. *laceratifolia* (PZ482414.1; this study), *F. altissima* (MW013819.1) [28], *F. benjamina* (NC_053836.1) [9], *F. carica* (NC_035237.1) [29], *F. curtipes* (NC_053833.1) [9], *F. erecta* (PP291718.1) [30], *F. lyrata* (NC_053838.1) [9], *F. microcarpa* (NC_053834.1) [9], *F. pumila* (ON257112.1) [31], *F. racemosa* (NC_028185.1) [32], *F. religiosa* (NC_033979.1) [6], *F. subpisocarpa* (NC_073014.1) [33], and *F. virens* (OP580936.1).

To quantify nucleotide diversity and identify candidate hypervariable regions, sliding-window nucleotide diversity (Pi) was calculated using the Nei–Li formulation [34] with a 600-bp window and a 200-bp step. At each alignment position, pairwise differences were computed among available A/C/G/T observations; positions with fewer than two valid observations after excluding missing data and ambiguous bases were excluded. To assess sensitivity to gapped, missing, or ambiguous observations, windows were retained and analyzed separately at minimum valid-site fractions of 0.5, 0.8, and 0.9 (Tables S5a–S5d). Alignment coordinates were mapped to the ungapped PZ482414.1 reference and annotated against its GenBank record. To avoid treating overlapping windows as independent candidates, the highest-Pi windows within each threshold set were ranked and collapsed into distinct annotated regions.

### 2.6. Phylogenetic Analysis

To infer plastid phylogenetic relationships, we analyzed 13 normalized *Ficus* plastomes together with *Morus indica* (NC_008359.1 [35]) and *Morus notabilis* (NC_027110.1 [36]) as outgroups, retaining IRb and removing IRa from each plastome to avoid duplicated phylogenetic signal. Four datasets were generated: whole single-IR (133,982 trimmed alignment columns), coding (68,431), non-coding (58,792), and partitioned (133,923). Coding, RNA, and non-coding site masks were derived from PZ482414.1 annotations and projected onto the single-IR alignment. Sequences were aligned with MAFFT v7.525 (--auto) and trimmed with trimAl v1.5.rev1 under the automated1 heuristic [37]. Maximum-likelihood phylogenies were then inferred in IQ-TREE v3.1.2 [38] under ModelFinder-selected models [39]: K3Pu+F+R4 for the whole dataset, TVM+F+I for the coding dataset, K3Pu+F+R3 for the non-coding dataset, and the best MFP+MERGE scheme for the partitioned dataset. Branch support was assessed with 1000 ultrafast bootstrap (UFBoot) replicates [40], and the morphology-based constrained topology was evaluated with an approximately unbiased (AU) test based on 10,000 resampling estimated log-likelihood (RELL) replicates [41]. The maximum-likelihood trees were inferred as unrooted topologies and subsequently rooted for display on the branch separating the two *Morus* outgroups from the sampled *Ficus* clade.

## 3. Results

### 3.1. Chloroplast Genome Organization

**Figure 2:**
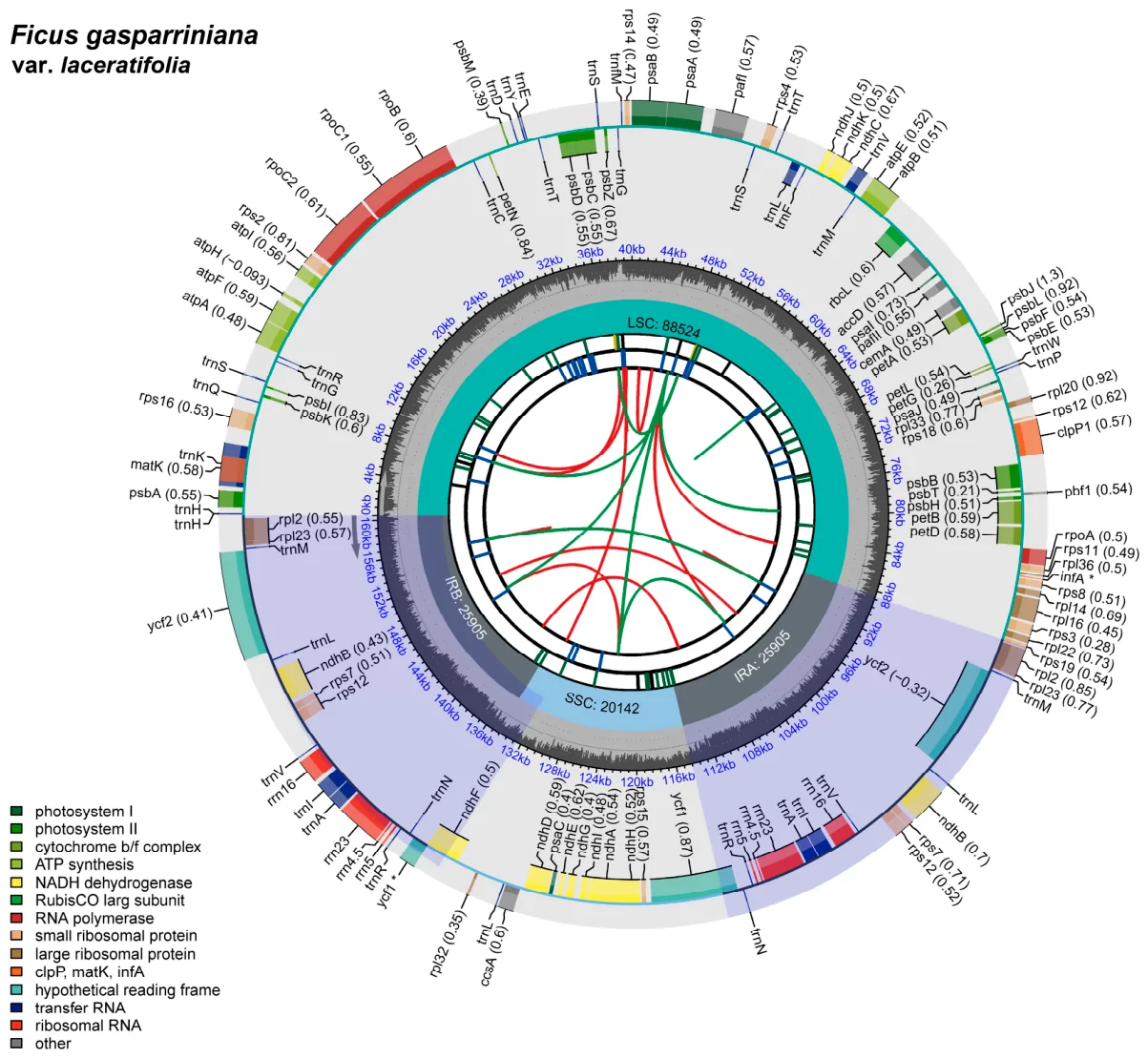
Chloroplast genome map of *Ficus gasparriniana* var. *laceratifolia*. The plastome is displayed in six concentric circles. From the inside out: dispersed repeats, tandem repeats, SSRs, LSC/SSC/IR boundaries, GC content, and annotated genes. Genes outside the circle are transcribed counter-clockwise, whereas genes inside the circle are transcribed clockwise. Asterisks indicate pseudogene features (*infA* and the truncated IR-associated *ycf1*); colors in the gene track correspond to the functional categories shown in the legend.

The complete chloroplast genome of *F. gasparriniana* var. *laceratifolia* (PZ482414.1) was 160,476 bp and exhibited the canonical quadripartite structure typical of angiosperm plastomes, comprising an 88,524-bp LSC, a 20,142-bp SSC, and two 25,905-bp IRs (Figure 2; Table 1). Primary-read coverage ranged from 113× to 9672× (median 2043×; mean 1999×), with every position covered by at least 100× (Figure S1). All four structural junctions were supported by MAPQ ≥ 20 spanning reads, unique spanning templates, and proper-pair fragment bridges, with minimum central 100-bp depths of 1315×–1806× (Table S8). Coverage across the IRa/LSC junction confirmed sequence continuity between positions 160,476 and 1. Comparison with an independently assembled plastome from SRR13279556 (160,447 bp) revealed 99.87% pairwise identity, with 160,330 identical alignment bases across a 160,537-column alignment, 56 single-nucleotide polymorphism (SNP) alignment columns, and 43 contiguous insertion/deletion (indel) events (Tables S7, S7a and S7b). These results confirm the reproducibility of the assembly across independent sequencing datasets.

### 3.2. SSRs and Long Repeats

The plastome contained 61 SSRs, comprising 54 mononucleotide repeats, six dinucleotide repeats, and one trinucleotide repeat, of which 10 formed compound loci (Table S1). A/T mononucleotide repeats accounted for 86.9% of all SSRs, consistent with the AT-rich composition of the plastome. These abundant short repeats provide candidate variable loci for future marker development and population-level studies in *Ficus*.

**Figure 3:**
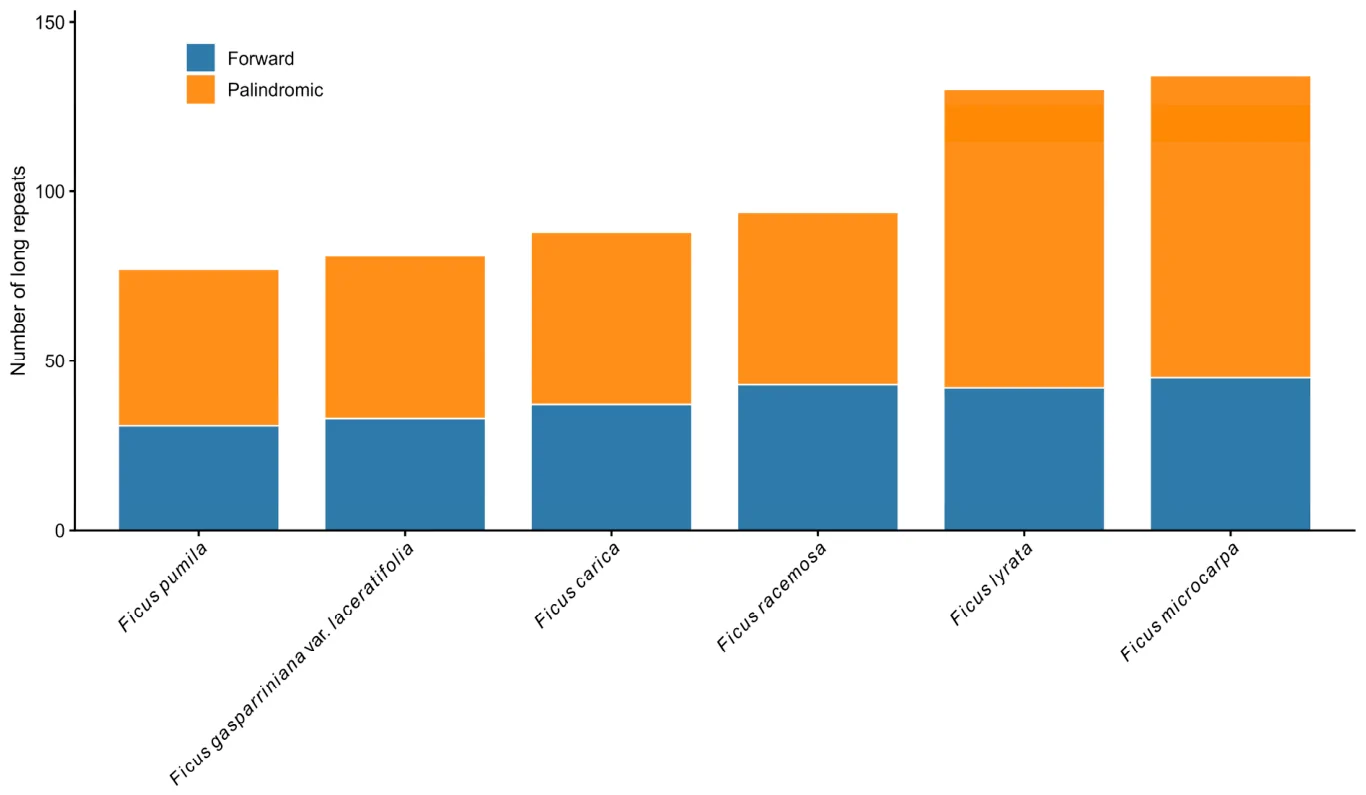
Long-repeat composition of six *Ficus* chloroplast genomes. Forward and palindromic repeats were detected using Vmatch with a minimum repeat length of 30 bp and a Hamming distance of 3.

Across the six sampled *Ficus* plastomes, long repeats comprised forward and palindromic classes (Figure 3; Table 2 and Table S2). *F. gasparriniana* var. *laceratifolia* contained 33 forward and 48 palindromic repeats (81 in total), the second-lowest count among the six taxa. This count was similar to those of *F. pumila* (77) and *F. carica* (88), but lower than those of *F. racemosa* (94), *F. lyrata* (130), and *F. microcarpa* (134). Palindromic repeats were more abundant than forward repeats in all six plastomes. Thus, the focal plastome combines an AT-rich microsatellite profile with relatively few long repeats compared with most sampled *Ficus* plastomes.

### 3.3. Relative Synonymous Codon Usage

**Figure 4:**
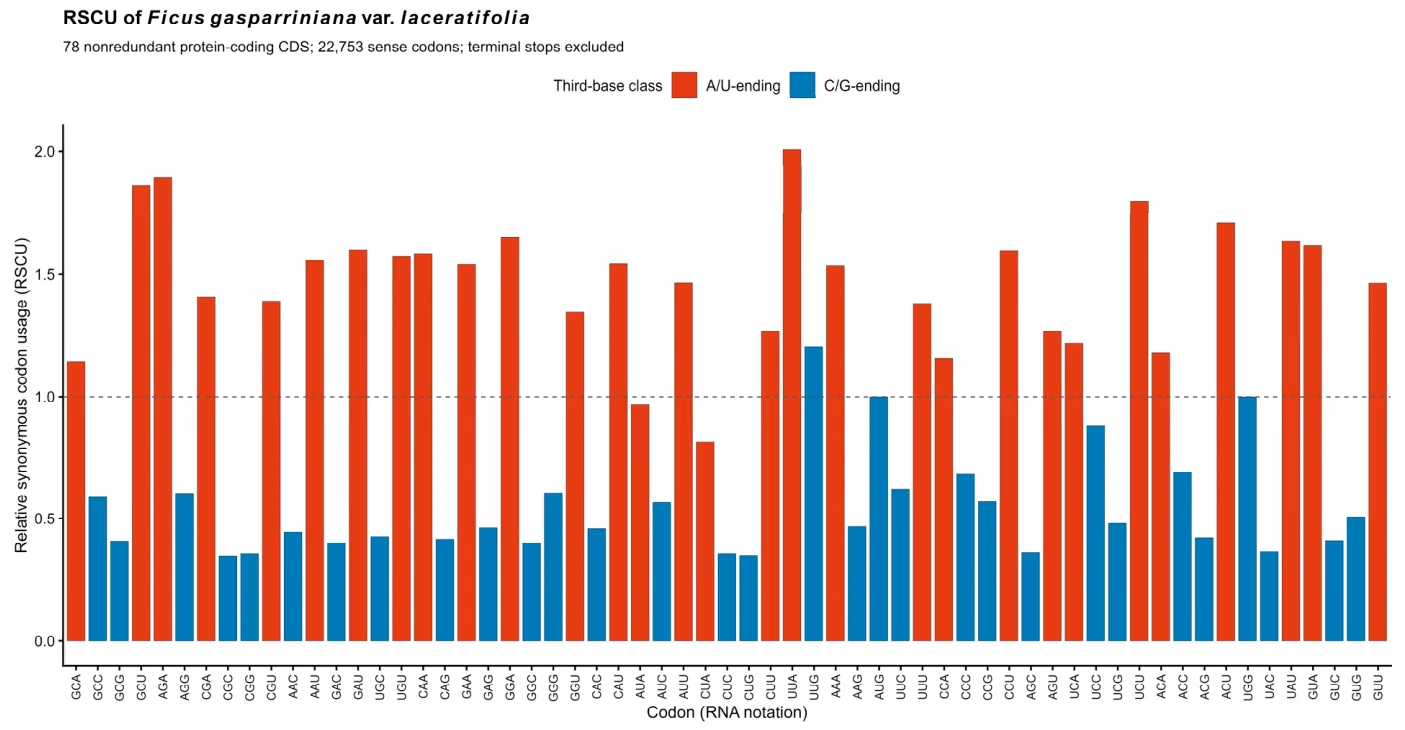
Relative synonymous codon usage (RSCU) in the *Ficus gasparriniana* var. *laceratifolia* chloroplast genome. Values are based on 78 nonredundant protein-coding CDS and 22,753 sense codons; terminal stops are excluded. The dashed line indicates RSCU = 1, and bar colors distinguish A/U-ending from C/G-ending codons. The all-copy sensitivity analysis is reported separately in Table S3b.

Analysis of 78 nonredundant protein-coding CDS (22,753 sense codons) identified 29 codons with RSCU > 1 (Figure 4; Table 3 and Table S3a). Of these 29 preferred codons, 28 ended in A or U; UUG was the sole G-ending exception. UUA (Leu; RSCU = 2.01) was the most overrepresented codon, indicating a pronounced preference for A/U-ending codons consistent with the AT-rich composition of the plastome. The all-copy sensitivity analysis (84 CDS; 26,199 sense codons) recovered the same overall pattern—29 preferred codons, of which 28 ended in A or U—even when duplicated IR copies were included (Table S3b).

### 3.4. IR Boundary Structure

The standardized IRscope comparison revealed conserved IR/SC junction configurations across six representative *Ficus* plastomes (Figure S4; Table S4). LSC, SSC, and IR lengths ranged from 88,110 to 88,824 bp, from 20,001 to 20,142 bp, and from 25,681 to 25,905 bp, respectively. The limited variation in regional lengths indicates that the focal plastome shares the conserved boundary structure of the sampled *Ficus* plastomes, with no large-scale IR expansion or contraction.

### 3.5. Hypervariable Regions

**Figure 5:**
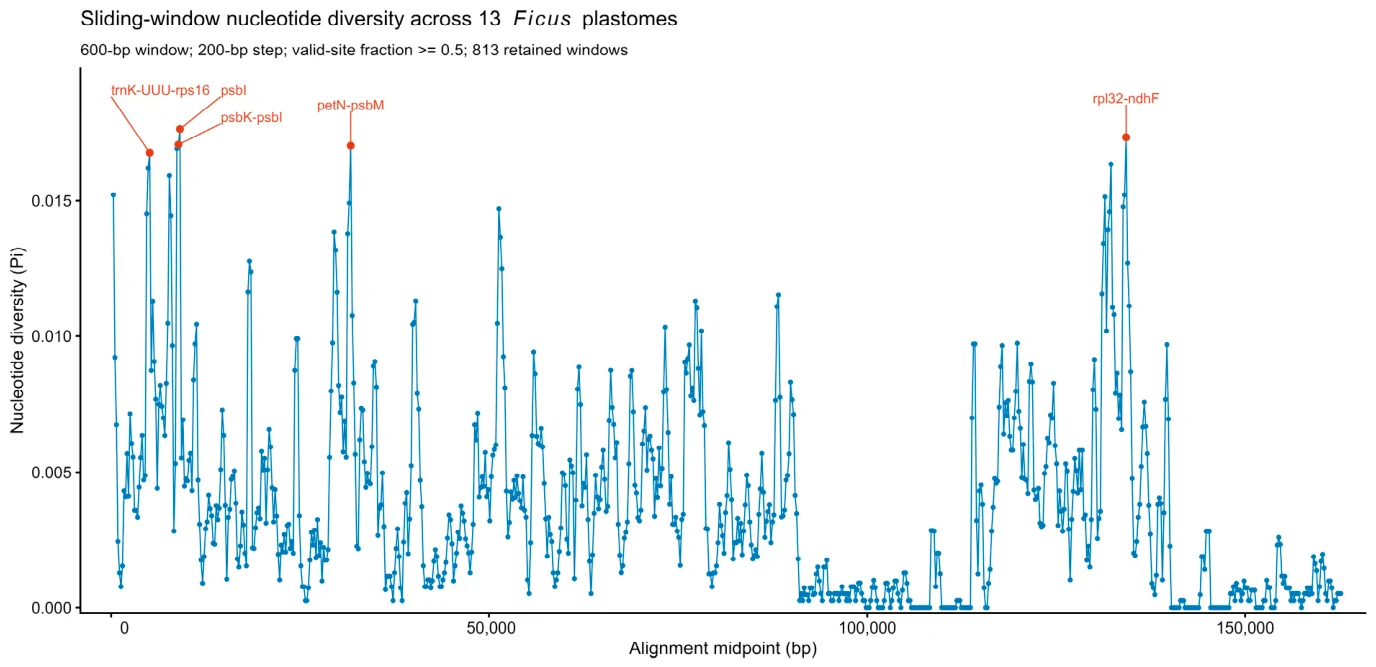
Sliding-window nucleotide diversity (Pi) across 13 SSC-normalized *Ficus* plastomes. Window size = 600 bp and step size = 200 bp; the plotted series retains windows with valid-site fraction ≥ 0.5 (813 windows). Red points and labels mark the five highest distinct annotated regions. Sensitivity tables retain 808 and 805 windows at valid-site fractions ≥ 0.8 and ≥0.9, respectively.

After start-position and SSC-orientation normalization, the alignment of 13 plastomes comprised 163,158 columns. Nucleotide diversity did not exceed 0.0177, with the maximum occurring in a window overlapping *psbI*. This low maximum indicates that sequence variation was limited overall and distributed among a few regions in the LSC and SSC rather than concentrated in a single genomic block. Other leading windows overlapped the *rpl32*–*ndhF*, *psbK*–*psbI*, *petN*–*psbM*, and *trnK*-UUU–*rps16* boundary regions (Table 4 and Table S6). The same leading regions were recovered across valid-site fraction thresholds of 0.5–0.9 (Figure 5; Tables S5a–S5d), demonstrating robustness to missing-data treatment and identifying them as candidate loci for future marker development.

### 3.6. Phylogenetic Placement

**Figure 6:**
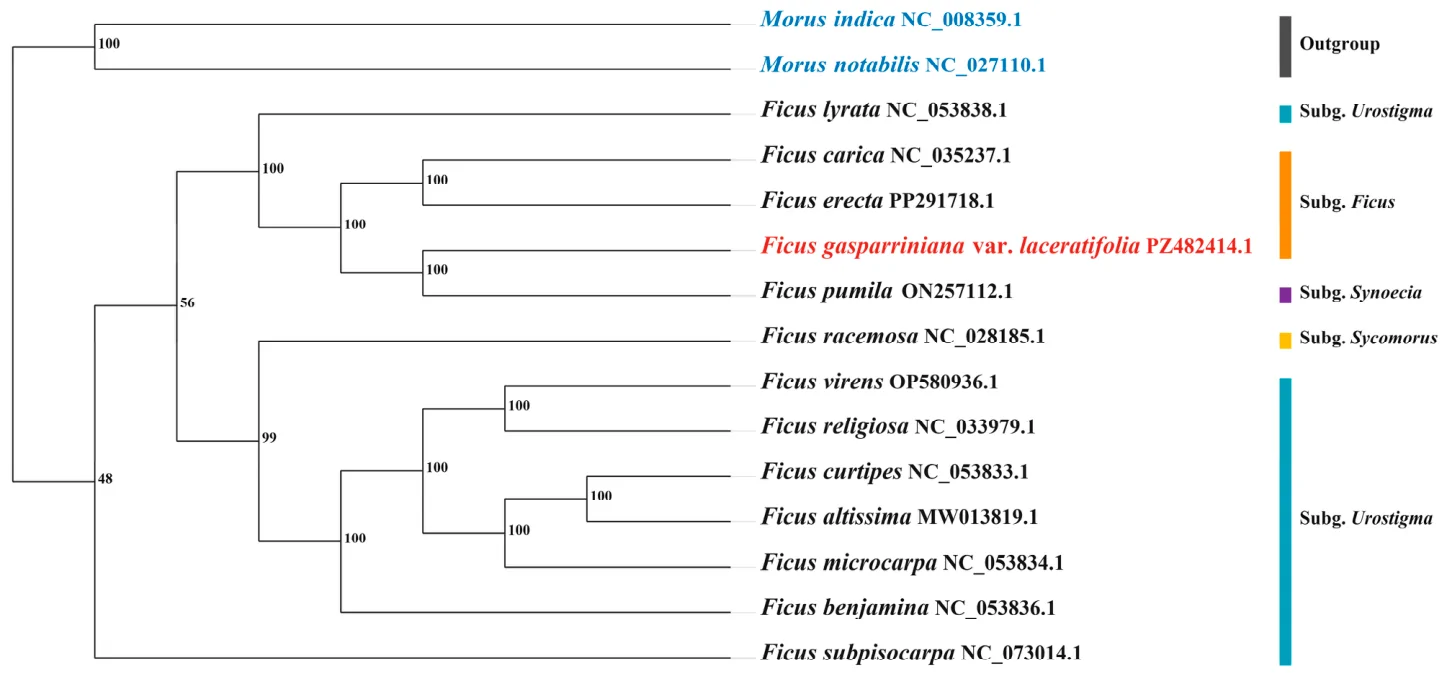
Maximum-likelihood plastid phylogeny based on the partitioned single-IR alignment (133,923 bp). The topology was inferred as unrooted and subsequently rooted for display on the branch separating *Morus indica* (NC_008359.1) and *Morus notabilis* (NC_027110.1) from the sampled *Ficus* clade; edge lengths are suppressed so that topology and support labels remain readable. UFBoot values are shown at internal nodes, accession numbers follow taxon names, and *F. gasparriniana* var. *laceratifolia* is highlighted. The whole, coding, non-coding and partitioned topologies are compared in Figure S5. The focal taxon is shown in red, the *Morus* outgroups in blue, and the colored bars indicate the subgenera identified in the legend.

Phylogenetic analyses of four complementary single-IR datasets (whole, coding, non-coding, and partitioned), displayed with *Morus indica* and *M. notabilis* as outgroups, each recovered a monophyletic sampled *Ficus* clade (Figure 6; the four topologies are compared in Figure S5). Contrary to its morphology-based placement in subg. *Ficus* alongside *F. carica* and *F. erecta*, *F. gasparriniana* var. *laceratifolia* grouped with *F. pumila*. The same focal unrooted bipartition was recovered in all four datasets with UFBoot = 100. AU tests rejected the morphology-based constraint in every dataset (Table S9). Although relationships elsewhere in the topologies varied among datasets, consistent recovery of this specific bipartition indicates a stable plastid signal for the placement of var. *laceratifolia*. Determining whether this pattern represents the organismal species tree and identifying the mechanism underlying the morphology–plastid discordance require nuclear genomic data.

## 4. Discussion

This study provides a complete chloroplast genome of *Ficus gasparriniana* var. *laceratifolia* and places it within a comparative plastome framework for *Ficus*. The plastome showed the canonical quadripartite structure, conserved gene content, stable IR boundaries, AT-rich SSR and codon-usage patterns, and several candidate variable regions, indicating a structurally typical yet informative plastome resource. Most notably, all four plastid datasets recovered var. *laceratifolia* together with *F. pumila* rather than with the morphologically expected members of subg. *Ficus*, and AU tests rejected the morphology-based constraint, revealing a clear discordance between morphological classification and plastid phylogeny.

### 4.1. Conserved Plastome Structure Supports the Reliability of the Inference

The morphology–plastid discordance is unlikely to be an artifact of assembly or sampling error. The focal plastome is circular, displays the canonical quadripartite structure, and is supported by deep junction-spanning coverage (Table S8), while an independently sequenced dataset (SRR13279556) yields a near-identical plastome (>99.8% pairwise identity; Tables S7, S7a and S7b). Moreover, all four single-IR datasets recovered the same focal split, and AU tests rejected the morphology-based constraint in every dataset. Together, these lines of evidence indicate that the conflicting placement reflects a genuine feature of the plastid genealogy rather than a sequencing or analytical artifact. The evolutionary and taxonomic interpretation of this discordance (Section 4.2 and Section 4.3) therefore rests on a reliable signal.

### 4.2. Plastid Lineage and Morphology-Based Classification Are Decoupled

The plastid grouping of *F. gasparriniana* var. *laceratifolia* with *F. pumila* is unexpected because the two taxa differ across multiple character systems, including growth habit, leaf morphology, and syconium traits. These morphological differences underlie their placement in different infrageneric groups under the Flora of China treatment [10] (Figure 1). The discordance therefore does not hinge on a single ambiguous character; rather, it shows that pronounced morphological differentiation can coexist with close plastid affinity in *Ficus*.

### 4.3. Alternative Mechanisms and Limits of Plastid-Only Inference

The observed morphology–plastid discordance is compatible with two principal evolutionary scenarios: chloroplast capture following hybridization and geographically localized introgression, or incomplete lineage sorting of ancestral plastid haplotypes. Previous *Ficus* studies have documented plastid–nuclear discordance and discussed ancient or localized introgression as possible explanations [6,7,42,43,44,45], placing the present result within a broader pattern of discordant phylogenetic signals in the genus. Distinguishing between these scenarios will require nuclear genomic data, coalescent species-tree inference, explicit network analyses, and broader geographic population sampling. The stable plastid signal for the focal placement provides a focused basis for testing these alternative hypotheses.

### 4.4. The Plastome Provides Candidate Markers for Future Ficus Studies

Beyond its phylogenetic implications, the assembled plastome offers practical resources for future *Ficus* research. The 61 SSR loci (Table S1) and five candidate variable regions (Table 4) identified here provide starting points for developing population-level markers and markers for species discrimination, extending established uses of chloroplast microsatellites and variable plastid loci in plant ecology and systematics [46,47]. Primer design, amplification testing, and taxon- and population-level sampling will be needed to evaluate these candidates for species authentication, phylogeography, and conservation genetics.

### 4.5. Limitations and Future Directions

This study has two main scope limitations. First, phylogenetic sampling is restricted to 13 *Ficus* plastomes, a small fraction of the roughly 800 species in the genus [1,2]; although the focal relationship is strongly supported within the current taxon set, its placement relative to the broader diversity of *Ficus* remains to be tested. Second, the plastome represents a single linked genealogy and therefore cannot by itself distinguish among the alternative evolutionary scenarios discussed above (Section 4.3). Broader taxon sampling and integration of nuclear genomic evidence will allow these alternatives to be tested, with the current plastome providing a reference point for those analyses.

## 5. Conclusions

This study presents the complete chloroplast genome of *Ficus gasparriniana* var. *laceratifolia* and establishes a structurally typical yet informative plastome resource for *Ficus*, including SSRs and five candidate variable regions for future marker development. Across four complementary single-IR datasets, plastid phylogenies consistently recovered the same focal unrooted bipartition uniting var. *laceratifolia* with *F. pumila* (UFBoot = 100), while AU tests rejected the morphology-based constraint in every dataset. This result adds to a growing body of evidence that pronounced morphological differentiation can coexist with close plastid affinity in *Ficus*, highlighting a decoupling between morphology-based classification and plastid lineage history. The plastome and focal phylogenetic signal provide a foundation for nuclear genomic and population-level studies of the processes underlying this discordance, future comparative studies, and conservation-genetic research.

## Figures and Tables

**Table 1:** Summary of complete chloroplast genome features of *Ficus gasparriniana* var. *laceratifolia*.

Feature	Value
Total sequence length (bp)	160,476
Large single-copy region (bp)	88,524
Small single-copy region (bp)	20,142
Inverted repeat region, each copy (bp)	25,905
Overall GC content (%)	35.90
LSC GC content (%)	33.56
SSC GC content (%)	28.91
IR GC content (%)	42.62
Total annotated genes	137
Unique genes/features	110
Total protein-coding genes	87
Total tRNA genes	42
Total rRNA genes	8
Annotated pseudogene features	2 (*infA* and truncated IR-associated *ycf1*)
Unique protein-coding genes	78
Unique tRNA genes	28
Unique rRNA genes	4

The plastome contained 137 annotated gene features representing 110 unique genes or gene features, including 78 protein-coding genes, 28 tRNA genes, and four rRNA genes (Table 1). Twenty-seven genes were duplicated within the IRs, consistent with the canonical quadripartite organization. Two pseudogene features, *infA* and a truncated IR-associated copy of *ycf1*, were detected and excluded from the RSCU analysis; the truncated *ycf1* represents the partial copy commonly generated at the SSC/IR boundary and is distinct from the intact *ycf1* coding sequence. Intron organization was similarly conserved: 11 unique genes contained *cis*-spliced introns (Figure S2), whereas *rps12* retained its characteristic *trans*-spliced structure with three unique exons, two of which were duplicated in the IRs (Figure S3). Together, these features are consistent with the structural organization typical of angiosperm chloroplast genomes.

**Table 2:** Long repeats detected in six *Ficus* chloroplast genomes using Vmatch.

Species	Forward Repeats	Palindromic Repeats	Total
*Ficus carica*	37	51	88
*Ficus gasparriniana* var. *laceratifolia*	33	48	81
*Ficus lyrata*	42	88	130
*Ficus microcarpa*	45	89	134
*Ficus pumila*	31	46	77
*Ficus racemosa*	43	51	94

**Table 3:** Ten highest-RSCU codons in the *Ficus gasparriniana* var. *laceratifolia* chloroplast genome based on 78 nonredundant protein-coding CDS and 22,753 sense codons. Duplicated IR copies are excluded from this analysis.

Amino Acid	Preferred Codon	Count	RSCU	Codon Ending
Leu	UUA	818	2.01	A/U-ending
Arg	AGA	423	1.89	A/U-ending
Ala	GCU	578	1.86	A/U-ending
Ser	UCU	505	1.80	A/U-ending
Thr	ACU	489	1.71	A/U-ending
Gly	GGA	643	1.65	A/U-ending
Tyr	UAU	710	1.63	A/U-ending
Val	GUA	511	1.62	A/U-ending
Asp	GAU	721	1.60	A/U-ending
Pro	CCU	375	1.60	A/U-ending

**Table 4:** Highest distinct annotated candidate regions from sliding-window Pi analysis across 13 SSC-normalized *Ficus* plastomes. Values are shown for the valid-site fraction ≥ 0.5 set.

Region	Alignment Window	PZ482414.1 Coordinates (bp)	Maximum Pi	Valid-Site Fraction	Region Type
*psbI*	8801–9400	8272–8853	0.01765191	0.971667	CDS
*rpl32–ndhF*	134,001–134,600	131,424–132,013	0.01734497	0.985000	CDS/IGS boundary
*psbK–psbI*	8601–9200	8075–8660	0.01707527	0.990000	CDS/IGS boundary
*petN–psbM*	31,401–32,000	30,602–31,139	0.01703297	0.933333	CDS/IGS boundary
*trnK-UUU–rps16*	4801–5400	4625–5175	0.01677383	0.958333	tRNA/CDS interval

## Data Availability

The complete chloroplast genome analyzed here as *Ficus gasparriniana* var. *laceratifolia* is available in GenBank under accession PZ482414.1 (indexed at the species level as *F. gasparriniana*). Raw reads from this study are available under BioProject PRJNA1472929, BioSample SAMN60525120, and SRA accession SRR38916853; independent reads used for sequence validation are under SRA accession SRR13279556 (BioProject PRJNA684963, BioSample SAMN17081732). The full analysis package, including scripts, alignments, and phylogenetic outputs, is archived on Zenodo (https://doi.org/10.5281/zenodo.21133164, which resolves to the latest version, currently https://doi.org/10.5281/zenodo.21440858). The *F. virens* sequence used in the diversity and phylogenetic analyses (OP580936.1) has associated reads under SRA accession SRR26662736 (experiment SRX22362799; BioSample SAMN38055522; BioProject PRJNA1034256). Variant support is detailed in Tables S7, S7a and S7b.

## Supplementary Materials

The following supporting information can be downloaded at: https://www.mdpi.com/article/10.3390/genes17080871/s1, Figure S1: Primary-read coverage across PZ482414.1, including LSC/IRb/SSC/IRa boundaries and a 100× reference line; Figure S2: Schematic map of *cis*-splicing genes; Figure S3: schematic map of the *trans*-splicing gene *rps12*; Figure S4: Standardized inverted-repeat junction comparison among six *Ficus* plastomes; Figure S5: Comparison of the whole, coding, non-coding and partitioned single-IR plastid phylogenies, inferred as unrooted and subsequently rooted with *Morus* for display; Table S1: SSR loci and compound formations; Table S2: Vmatch long-repeat summary; Table S3a: Codon counts and RSCU values for 78 nonredundant protein-coding CDS; Table S3b: All-copy RSCU sensitivity analysis including duplicated IR CDS; Table S4: Standardized inverted-repeat boundary and region-length comparison; Tables S5a–S5d: Raw and thresholded 600-bp sliding-window Pi results at valid-site fractions 0.5, 0.8 and 0.9; Table S6: Pi peak annotations and candidate-region status; Table S7: Assembly, alignment and read-support summary with separate alignment-defined and read-supported units; Table S7a: Alignment-defined SNP columns and read-support evidence; Table S7b: Contiguous indel events and read-support evidence; Table S8: Junction coordinates, MAPQ ≥ 20 spanning reads, unique spanning templates, proper-pair fragment bridges and central 100-bp minimum depth across all four plastome boundaries; Table S9: Alignment characteristics, support for the focal unrooted bipartition, and AU test results across four single-IR plastid datasets.
