# Supplementary material for: The Complete Chloroplast Genome of Ficus gasparriniana var. laceratifolia Reveals Discordance Between Morphology-Based Classification and Plastid Phylogeny
Source: Genes (Basel). 2026 Jul 26;17(8):871. doi: 10.3390/genes17080871 (PMC13512103; doi:10.3390/genes17080871)
Supplement: Supplementary file 1 [file genes-17-00871-s001.zip › Supplementary_Figures_S1-S5_v11.43.pdf]

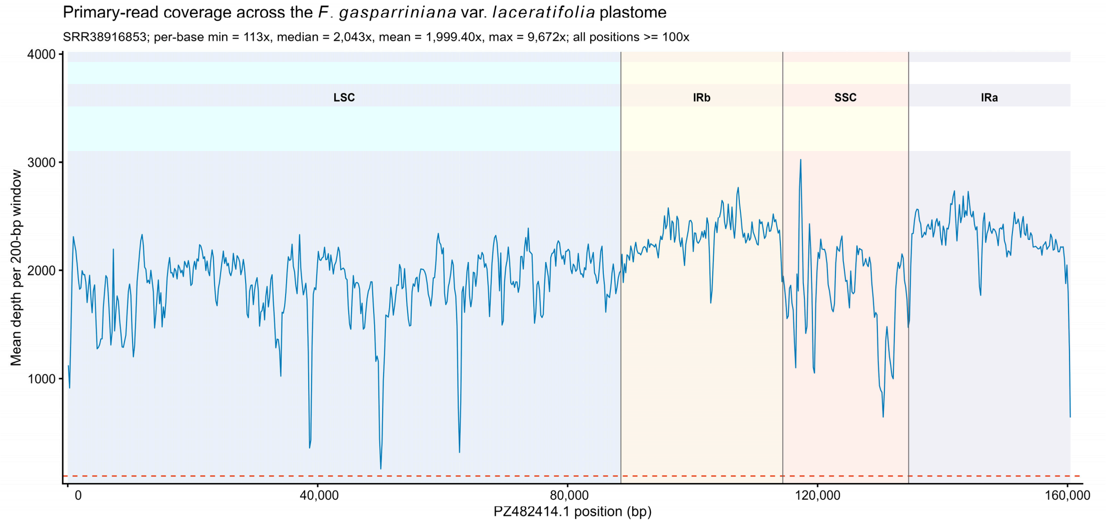

Supplementary Figure S1. Primary-read coverage across the complete PZ482414.1 plastome associated with the *Ficus gasparriniana* var. *laceratifolia* voucher. Mean depth is shown in non-overlapping 200-bp windows; shaded blocks identify LSC, IRb, SSC and IRa. The dashed line marks 100×. Across 160,476 reference positions, primary-read depth ranged from 113× to 9,672× (median 2,043×; mean 1,999.40×), and every position met the 100× threshold.

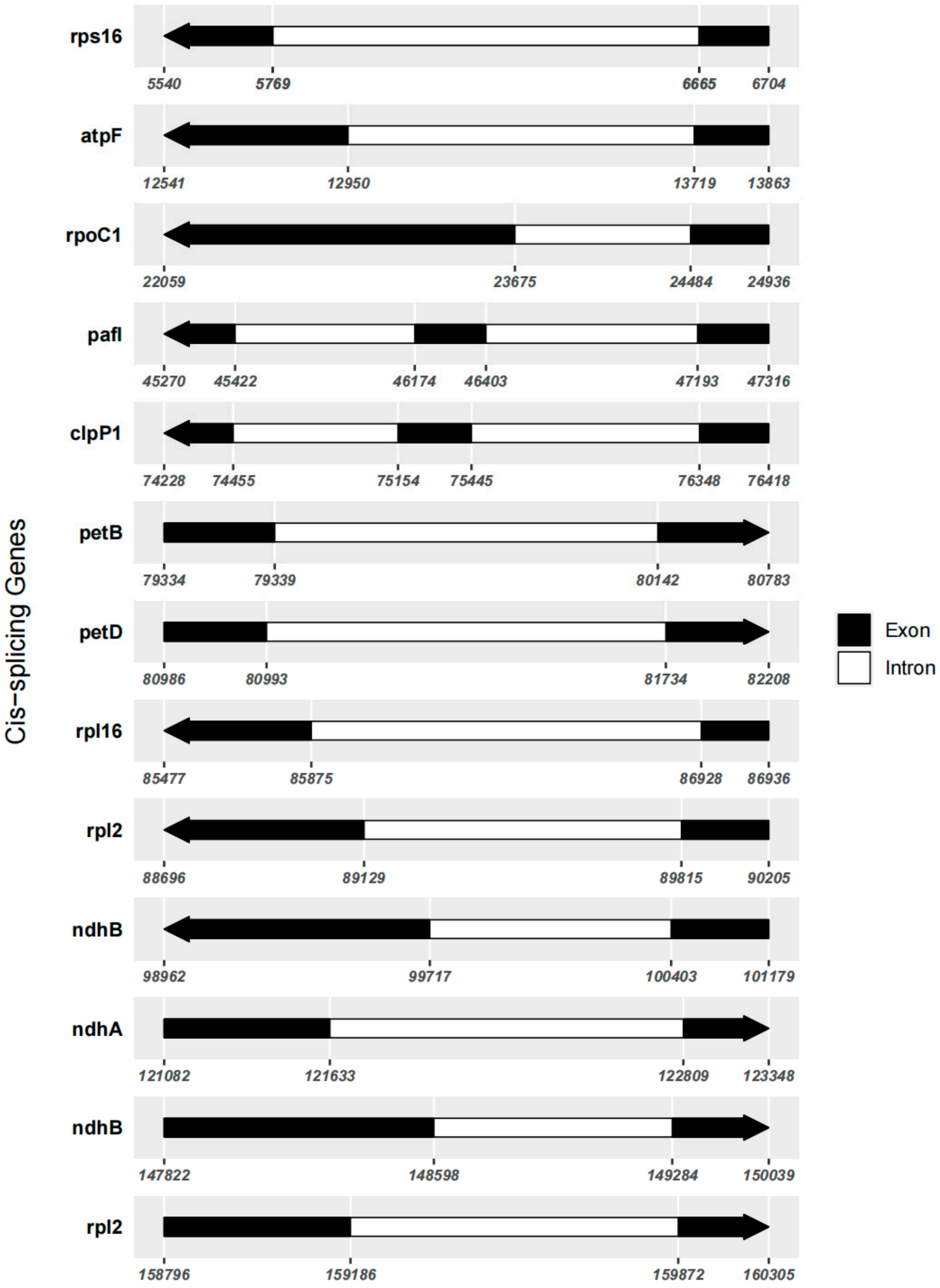

Supplementary Figure S2. Schematic map of the *cis*-splicing genes in the chloroplast genome of *Ficus gasparriniana* var. *laceratifolia*. Gene names appear on the left side, and the corresponding structures are displayed on the right. Exons and introns are shown in black and white, respectively. Arrows indicate transcriptional direction.

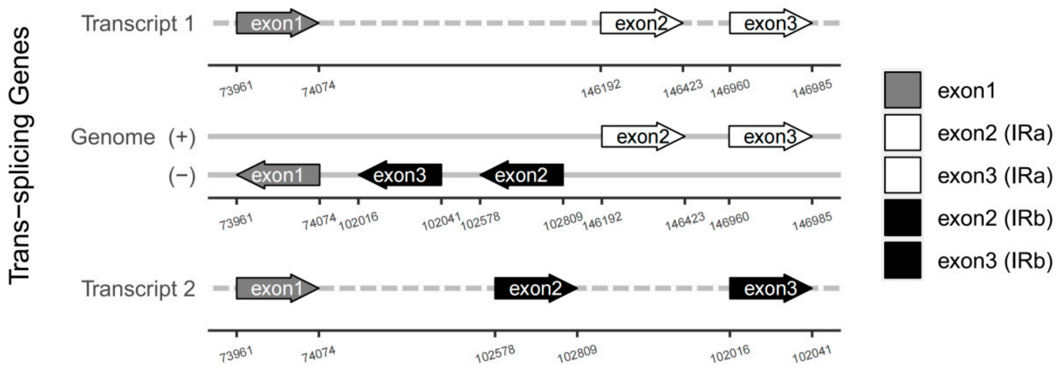

Supplementary Figure S3. Schematic map of the *trans*-splicing gene *rps12* in the chloroplast genome of *Ficus gasparriniana* var. *laceratifolia*. The gene has three unique exons; exons 2 and 3 are duplicated because they occur in both IR regions.

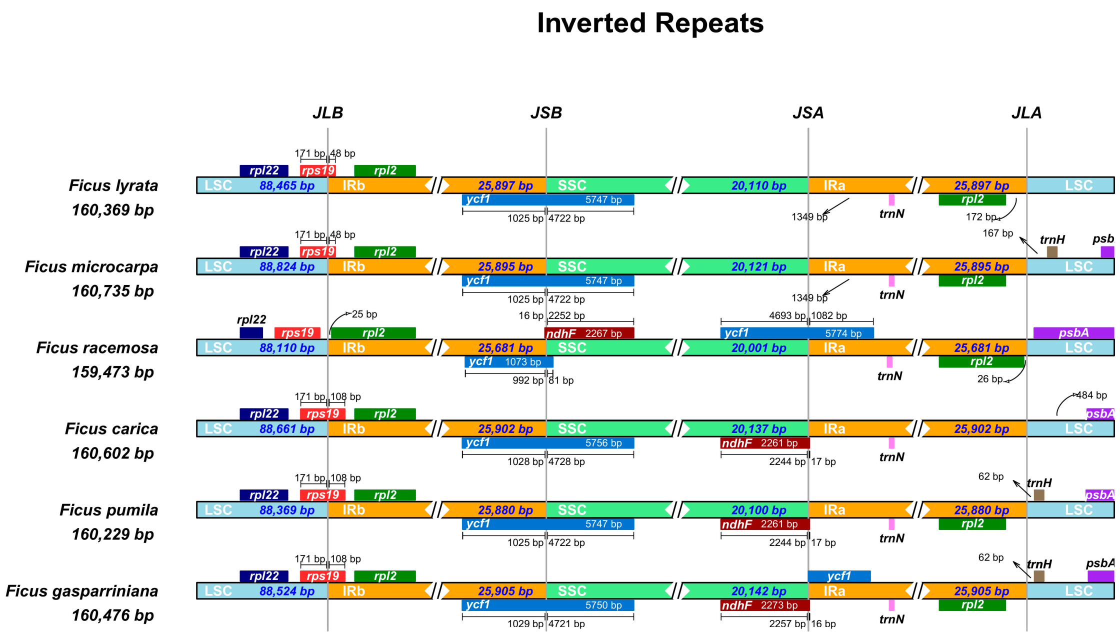

Supplementary Figure S4. Comparison of inverted-repeat junctions among six *Ficus* chloroplast genomes, including the focal *F. gasparriniana* var. *laceratifolia* plastome. Junctions are shown for LSC/IRb (JLB), IRb/SSC (JSB), SSC/IRa (JSA), and IRa/LSC (JLA). IR boundaries were visualized using standardized GenBank records with self-BLAST-verified `repeat_region` annotations; nucleotide sequences and accession identities were not modified.

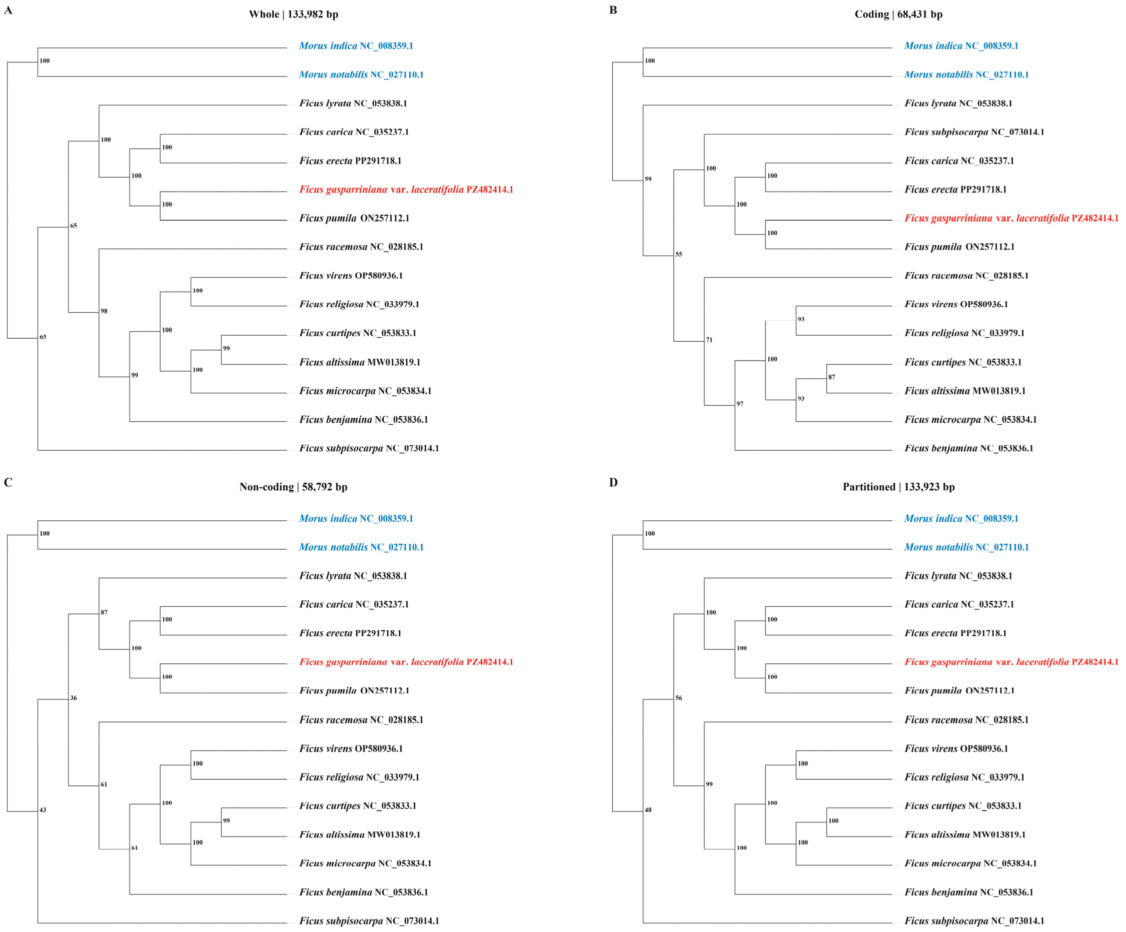

Supplementary Figure S5. Maximum-likelihood plastid phylogenies based on four single-IR datasets: (A) whole, 133,982 bp; (B) coding, 68,431 bp; (C) non-coding, 58,792 bp; and (D) partitioned, 133,923 bp. Trees are rooted for display with *Morus indica* (NC_008359.1) and *Morus notabilis* (NC_027110.1), and edge lengths are suppressed for topology comparison. UFBoot values are shown at internal nodes, accession numbers follow taxon names, and *F. gasparriniana* var. *laceratifolia* is highlighted.
